# Poor perception of chronic kidney diseases and its influencing factors among diabetics patients

**DOI:** 10.1038/s41598-022-09354-y

**Published:** 2022-04-05

**Authors:** Shamsul Azhar Shah, Haryati Anuar, Abdul Halim Abdul Gafor, Nik Nairan Abdullah

**Affiliations:** 1 Department of Community Health, Faculty of Medicine, Universiti Kebangsaan Malaysia, Kuala Lumpur, Malaysia; 2 Department of Pharmaceutical Biology, Faculty of Pharmaceutical Sciences, UCSI University, 56000 Kuala Lumpur, Malaysia; 3 Department of Public Health Medicine, Faculty of Medicine, Universiti Teknologi MARA, Sungai Buloh, Selangor Malaysia

**Keywords:** Diseases, Health care

## Abstract

*Purpose* We aimed to determine predictors of chronic kidney disease (CKD) prevention among patients with diabetes. *Method* A cross-sectional study was conducted on 1000 selected respondents based on socio-demographic, socio-economic, general CKD perception knowledge, self-monitoring advocacy, preventive behavior, treatment compliance, and psychosocial factors. Using multiple logistic regression, variables and their association with impaired perception of CKD prevention were analyzed. *Results* Overall, 74% had poor perception regarding CKD prevention (68.7% of men and 31.3% of women). In multivariable analysis, those with weak illness identity fear were two times more likely to have poor perceptions (95% CI 1.563–3.196, *p* < 0.001). Respondents with weak medical practice (AOR = 2.33, 95% CI 1.609–2.381, *p* < 0.001) and weak cooperation (AOR = 1.563; 95% CI 1.099–2.224, *p* < 0.001) were more likely to have poor perceptions on CKD prevention. Concerning poor perception, significant predictors were self-employment, housewives, working in private jobs, weak knowledge on clear glycosuria, sleep problems, print media, digital media, illness identity fear, weak medical practice, and weak co-operation factors. *Conclusion* Media support is crucial for supporting and improving positive views regarding CKD knowledge. Interventions to reach people with limited awareness on CKD prevention, lower socioeconomic status, and poor social support may improve identification of patients with early-stage CKD. Particular care should be taken to recognize and provide necessary services regarding the early detection of CKD.

## Introduction

Chronic kidney disease (CKD) is a leading cause of morbidity and mortality worldwide. It is related to several unfavorable symptoms and comorbidities^1^. The goal of CKD clinical management, including blood pressure, glycemic regulation, diet, and lifestyle changes, is to preserve renal function alongside treatment and prevention^2^. The therapeutic goals assess various patient clinical needs with the highest being the regular drug burden for all chronic conditions^3^. However, therapeutic objectives are difficult and non-compliance among patients is high^4^. Furthermore, in the event of non-adherence, ways to enhance self-management were ultimately shown to be associated with a high risk of causal death^5^.

In Malaysia, CKD rates have risen dramatically over the years. Though the government has made several efforts to prevent CKD, the number of deaths has increased dramatically^1^. Factors affecting disease knowledge among diabetic patients need to be studied extensively so that community involvement, via prevention programs, become effective.

The Ministry of Health (MOH) in Malaysia plays an important role in implementing various CKD programs and routines. Many projects that require community participation, such as the COMBI example, have been introduced in recent years^6^. Unfortunately, community involvement was only high in the early phases and declined over time. Due to a lack of motivation or other factors, people became less interested in participating in the program. There is therefore a need to study how community involvement can be encouraged within an effective and year-round prevention program^7,8^.

Our aim was to develop predictive models for the health determinants of patients with poor perception of actions that can reduce the risk of CKD. The study was to measure patient perceptions regarding CKD prevention and the factors contributing to public perceptions. Such contributing factors have a significant influence on public perceptions and can affect both negative and positive attitudes toward CKD. It has been emphasised in the literature that socio-economic position, both individual and neighborhood, acts as the primary exposure to health perceptions^9^.

## Method

This cross-sectional study was conducted at the Primary Care Clinic University Kebangsaan Malaysia Medical Centre, also known as Canselor Tuanku Mukhriz Medical Centre. It is located in Bangunan Dwitasik Plaza, Bandar Tasik Permaisuri, and Cheras Kuala Lumpur. The population sample included 1300 patients with diabetes mellitus from several units in the primary care clinic. The first 300 patients were involved in the construction and validation of questionnaires, while 1000 patients were included in the analysis study. This study was conducted between March and June 2016. Recruitment was conducted through an interview with those respondents who met criteria to participate in this research. In the waiting area, the interview session was held by contacting those respondents who agreed after completing the informed consent form and were promptly informed of the study's objective.

### Participants and data collection

The eligibility criteria included patients at least 18 years old, those diagnosed with diabetes mellitus, Malaysian citizens, and those who understand either English or Malay. Patients with CKD, mental illness, or illiteracy were excluded from this study.

### Sample size calculation

From the literature review, the most appropriate sample size was considered based on the ratio of the likelihood variable, with a plus 10% of those counted for 824 patients. However, to fulfil the construct of the questionnaire that needed to be based on 65 items with a range of responses, another 300 patients were needed for validation purposes for the study to run. The total sample size of 1300 patients were decided upon by following the inclusion and exclusion criteria during the sample selection period. Therefore, 1000 patients are sufficient to run this study.

### Instruments

The research instrument used in this study was a set of self-administered questionnaires that had been validated before conducting the study. The thorough investigation of all the models revealed the Health Belief Model (HBM) domains were the most powerful and fulfilled the criterion for health preventive behavior. Several of the behavioral models used attempted to predict the medication noncompliance of patients related to health behavior (Anuar et al. 2020). The Chronic Kidney Disease Perception Scale (CKDPS) was designed to fill the gap in the perception of CKD among patients with diabetes mellitus in high-risk groups. The CKDPS questionnaire included: Part A: Socio-demographic and socio-economic; Part B: general knowledge of CKD; Part C: socio-psychology; and Part D: CKD Perception^10^. All questionnaire topics were reviewed in advance for their appropriateness regarding the research goals, data collection style, and sample population cultural diversity (Anuar et al. 2020).

### Definition for operating variables

#### The dependent variables

##### Level of perception of CKD prevention

The respondents’ level perception consists of five types of domains, perceived benefit, perceived barrier, perceived susceptibility, perceived severity and perceived cue to action. The scores for each of these domains are summed into one cumulative score. The cumulative score is categorized into two: A score of 223 and above is categorized as a good perception while scores less than 223 is categorized as poor perception. The category of perception was determined by selecting a score value representing 25% of the highest value as good, while 75% of the lowest value was considered as poor. The scores were in the range of 163–489. Value at the top of 25% and lowest at 75% on the graph score distribution is the determining point.

##### Factors in the health belief model

*Perceived benefits* How CKD preventative action are perceived, whether positive or negative, has an influence on disease management. Six questions covered the cost/benefit domain using the Likert scale with response options of one to ten where one = strongly disagree and ten = strongly agree.

*Perceived barriers* Patients’ attitudes to obstacles to the prevention of CKD in long-term conditions, what the respondents perceive as barrier to them following the CKD prevention in long term. Eight questions covered this domain using the Likert scale with response options of one to ten, where one = strongly disagree and ten = strongly agree.

*Perceived susceptibility* Respondent’s susceptibility indicates whether a positive identity is preserved among high level diabetes patients and there were eight questions in this domain using the Likert scale with response options of one to ten, where one = strongly disagree and ten = strongly agree.

*Perceived severity of illness* The severity of their diabetes can increase respondents’ perception of the usefulness of monitoring their health conditions. This again used the Likert scale response options of one to ten, where one = strongly disagree and ten = strongly agree.

*Perceived cues to action* Behaviors can be influenced by participation in various areas of life. Five questions on this domain were included using the Likert scale response options of one to ten, where one = strongly disagree and ten = strongly agree.

### The independent variables

Socio-demographic and socioeconomic factors.

**Age**: The respondent's age is based on the date of birth stated in the questionnaire
**Gender**: Gender of respondents: either male or female
**Religion**: Religious affiliations of the respondents whether Islam, Buddhism, Christianity or other religions
**Race**: The respondents were asked whether they were Malay, Chinese, Indian or other belonged to another group
**Marital status**: Marital status, in this study, has four classifications

- **Married**
  means legally married to a person of the opposite sex not separated or divorced)
- **Separated or divorced**
  Refers to divorced from a member of the opposite sex through due process, either on-going complete
- **Widowed**
  Refer to a woman or men who lost her spouse through death or unmarried women after the death of their future husband
- **Never married**
  Refers to unmarried people

**Occupation sector**: Type of work of the respondent, either with government bodies, private companies or self-employed an unemployed as others in open-ended answer
**Level of education**: The highest education level of respondents, either did not attend school, reached primary school, high school graduation, college graduation, college or university level
**Household income**: The respondent’s monthly household income (from everyone in the same house), including salary, allowances or income from an additional part-time job (if any). Respondents should indicate the estimated amount of their household monthly income on the questionnaire
**Individual income**: The respondent personal income including salary, allowances or income from another part-time job (if any)
**Type of insurance**: Accident, medical, life, education and/or income replacement
**Monthly payment**: Monthly insurance premium payments paid to cover their policies
**Insurance sum issued**: The total lump sum money that the insured person will receive if the worst happens

### Knowledge of diabetes mellitus

*Knowledge of CKD* General information about CKD prevention known by diabetic patients. Categorical variable, measured by six domains: socio demographic, socio economic, self-monitoring advocacy, treatment compliance, preventive behavior and psycho-social behavior.

### Psychosocial individual factors

#### Illness identity and fear of CKD

The level of illness identified that could lead to CKD as perceived by diabetic patients using medication provided by the hospital rather than complimentary or alternative medicines, their motivation to decrease anxiety and depression, taking their physician’s advice, positive reinforcement for better compliance, improving compliance related to financial incentives, continuous (or not) support from family members and knowledge of family members which increase a positive attitude towards CKD prevention either in terms of the community or health personnel. Illness identity is a continuous variable according to the symptoms experienced and reported by patients. It is measured by seven questions concerning identification and related concerns about the prevention of CKD using the Likert scale with response options of one to ten, where one = strongly disagree and ten = strongly agree. The category was determined by selecting a score value representing 25% of the highest value adequate, while 75% of the lowest value was considered as weak. The score was in the range of 7–70. Value at the top is 55–70 as adequate and 7–54 as weak score distribution is the determining point.

#### Timeline and motivation of CKD perception

Timeline and motivation in CKD prevention practices when it is understood that CKD can lead to death, involving monitoring Blood Pressure, blood glucose and sugar levels to improve the quality of life of the patients by removing the fear of developing CKD by taking control over their illness and accepting the process diagnosis and monitoring and that lab tests to identify the level of illness can help reduce the chances of getting CKD. There are nine questions covering the timeline and motivation domain using the Likert scale of response options one to ten, where one = strongly disagree and ten = strongly agree. The category was determined by selecting a score value representing 25% of the highest value adequate was in the range of 70–90, while 75% of the lowest value was considered as weak in the range on 9–69 based on the graph score distribution. The score were in the range of 9–90.

#### Patients’ medical responses to the feelings about CKD?

This domain covered what diabetic patients felt about their blood glucose levels and blood pressure when they visited nearby clinics if it was higher than reading, if they took their medication only when they fell sick, or if they believed that increasing the dosage would reduce their compliance with CKD prevention. Four questions were concerned with the medication behavior domain, using the Likert scale with response options of one to ten, where one = strongly disagree and ten = strongly agree. The category was determined by selecting a score value representing 25% of the highest value adequate while 75% of the lowest value was considered as weak. The score was in the range of 4–40. Value at the top is 31–40 as adequate and 4–30 as weak score distribution. 

#### Cooperation relating to how they perceived CKD

The patient’s reaction to lifestyle modifications due to the worse symptoms if they were confident the treatment it could increase their chances of developing CKD. The implications of the diagnosis and self-management can work together if rewarded and fully supported by the government hence can motivate the patients to fully participate in the campaign. There are six questions in this domain using the Likert scale response options one to ten where one = strongly disagree and ten = strongly agree. The category was determined by selecting a score value representing 25% of the highest value adequate, while 75% of the lowest value was considered as weak. The score was in the range of 6–60. Value at the top is 47–60 as adequate and 4–46 as weak score distribution is the determining point.

#### Self-monitoring advises

In this study, general knowledge on self-monitoring advocacy such as illness diseases, physical symptoms or indicators, self-care on CKD sign and symptoms toward perception of CKD prevention consists of 21 questions toward perception of CKD prevention. The category was determined by mean score value at the top is 16–21 was considered as adequate while 0–15.75 as weak score distribution. The following content for self-monitoring advocacy as per below:

**Monitor glucose levels**: Glucose level had been monitored daily by the patient
**Diabetics characteristics**: The patient was aware if what signs of diabetes to look out for in everyday life
**Physical symptoms to CKD**: Knowledge of diabetic patients on physical indicators that can contribute to CKD
**Self-care on CKD sign and symptoms**: Knowledge of diabetic patients on self-care on sign and symptoms that can contribute to CKD

#### Compliance with treatment

The domain covered of 8 questions in compliance with advised treatment as one of other predictors, such as laboratory tests and prevention practices for the interventions to avoid CKD. The category was determined by mean score value at the top is 7–8 was considered as adequate while 0–6 as weak score distribution. The following content for treatment compliance as per below:

**Laboratory test**: General laboratory tests for EGFR, ACR and Ultrasound help prevent CKD
**Prevention practices**: Daily lifestyle practices that can reduce the chances of getting CKD

#### Preventive behavior

The preventive behavior consists of 6 questions: input in health information and participation in health program that associated with perception among diabetic patients. The category was determined by mean score value at the top is 5–6 was considered as adequate while 0–4.5 as weak score distribution. The following content for self-monitoring advocacy as per below:

### Health program

The primary sources, the situation which the majority of patients were referred to a physician that gather the information on healthy lifestyle changes and to adopt with the new norm on healthy behavior.

#### Health information inputs

Sources of the CKD information that has been received by patients.

#### Participation in health programs

Volunteering to be involved in health programs carried out by the health organization.

### Data analysis

Data were analyzed using SPSS version 21 (SPSS Inc, Cary, NC, USA). The data were calculated descriptively to illustrate the study population and their perceptions. Bivariate analyses were conducted to examine the relationship between demographic, socioeconomic status, and general knowledge of CKD, as well as either good or poor knowledge regarding CKD prevention. There are 3 models involved in hierarchical logistic regression. A multiple logistic regression analysis was then used to identify any independent correlates to poor perception among patients with diabetes mellitus, forcing interest variables into the model. The methods of interaction were thoroughly examined.

### Ethics approval and consent to participate

The study was performed in accordance with the ethical standards as laid down in the 1964. Declaration of Helsinki and its later amendments or comparable ethical standards. This study was approved by the Ethics committee of the National University of Malaysia, UKM PPI/111/B/JEP-2017 420. Written consent was obtained from all participants.

## Results

### Development and validation of questionnaires

The scale was distributed for construct validation purposes to 300 diabetic patients in Primer clinic. Overall, the respondents were aged 31–87 years with an average of 62.24 and standard deviation of 9.3. There were more female respondents, 53.7% compared to 46.3% of male respondents. In terms of work, many of them were 75.3% housewives, most of whom were 51.3% Malay. 73% earned primary or secondary education, 53.3% being Islam. Based on the reports, almost 90% of diabetic patients have no health, career, employment, injury, or income-related benefits.

The total deleted items are four of 65 and 61 items representing eighteen factors. The eighteen factors construct, however, not only exhibited an inappropriate reliability alpha of 0.7, but the things representing nine constructs suggesting the socio-psychology of four constructs, namely illness identity and fear, timeline and motivation, medical practice and cooperation against CKD perception. The other 5 constructs based on perceived benefit, perceived barrier, perceived susceptibility, perceived severity, and perceived action cue. More PCA was then performed. Most items from the eighteen constructs merged produced a nine-construct solution with items loaded on the same construct. The nine components above 1.0 with overall scale reliability were 0.789 and 0.806 with good sub-scale reliability from 0.8 to 0.9.

The convergent discriminatory validity and reliability of each domain was determined for both AVE and CR. The AVE values ranged between 0.56 and 0.74 and were above the cut-off value of 0.5, indicating an acceptably convergent validity for all five domains. This finding shows that all domains were well discriminated against. The Cronbach alpha of all domains ranged from 0.76 to 0.91 and were comparable with their respective Cronbach α. The internal consistencies of each domain were strong.

Calculated to assess the convergent / discriminant validity and reliability of each domain, the AVE and CR as per the AVE values were 0.57–0.79, and all were above the cut-off value of 0.5 and demonstrated a reasonable convergent validity for all 5 domains. This finding showed that all domains were of strong racial quality. All domains of Cronbach alpha ranged from 0.90–0.97 and were comparable with their respective Cronbach alpha and showed that the internal consistencies of the domains were strong.

### Study population

In this study, the majority were Malaysians aged between 31 and 90 years, with a mean age of 63 years (Net males (68.7%) and females (31.3%) with a 2:1 ratio). Most respondents were married, only 0.7% single, and 0.5% were widows. Regarding educational attainment, 4.7% were students, while Sijil Pelajaran Malaysia (SPM) was 80.4%. Almost 69.6% were pensioners or housewives, and 30.4% worked in government, private, business, or were self-employed.

The median income of individual respondents was RM1000, while the income of the household per unit was RM3000 per month. We found that health and life insurance contributed only 17.9% of the total population. Most (80.2%) did not have health insurance (see Table 1).

**Table 1:** Demographic variables and socioeconomic status contributing to CKD.

Variables		Number, n	Percentage, %	Mean (Sd)	Min Max
Age	31–40 41–50 51–60 61–70 71–80 81–90	13 96 281 401 207 2	1.3 9.6 28.1 40.1 20.7 0.2	62 (9.10)	31 62
Gender	Male Female	687 313	68.7 31.3		
Marital Status	Married Divorce Widow/widower Unmarried	988 0 5 7	98.8 0.0 0.5 0.7		
Religion	Islam Buddha Hindu Christian Others	513 387 88 12	51.3 38.7 8.8 1.2		
Race	Malay Chinese Indian Others	513 393 94	51.3 39.3 9.4		
Occupation Sector	Government Private Business Self-employed Housewife/ husband Others	37 69 89 109 696	3.7 6.9 8.9 10.9 69.6		
Education level	Degree/Master /PhD Diploma /Certificate Secondary school Never attend school	47 70 804 79	4.7 7.0 80.4 7.9		
Monthly income(median) -Individual -Household				1991.90 3150.00	300 7500 400 10,000
Insurance	Health Life Education Self-accident Income Replacement	179 179 0 101 0	17.9 17.9 0.0 10.1 0.0		
Monthly payment	No premium RM50-RM400 RM401-RM800 RM801-RM1500 Above RM 1500	720 277 2 1 0	72.0 27.7 0.2 0.1 0.0		

*Mean (Sd).

Table 2 provides an overview of the level of understanding regarding CKD prevention, knowledge of CKD, and social support. Perception levels had broad standard deviations, implying a significant amount of variability within the sample. The analysis also revealed that 74% of respondents had a poor perception of CKD prevention, while 26% had good perception.

**Table 2:** Predictors on general knowledge of CKD.

Question	n	(%)
**Knowledge**		
Do you have other diseases besides diabetes?	Yes: 886	88.6
Have you ever been told by a physician that you have diabetes?	Yes: 966	96.6
Are you trying to get health information?	Yes: 663	66.3
Would you like to participate in a health program organized by the hospital?	Yes: 579	57.9
Do you monitor glucose levels?	Yes: 641	64.1
**Diabetic Characteristics**		
High levels of glucose in blood	Yes: 376	37.6
Clear glycosuria	Yes: 30	30.0
Ketonuria	Yes: 15	15.0
Kidney stone	Yes: 17	17.0
**Respondent’s Resources**		
Mass media: Television, Newspaper, Magazines	Yes: 452	45.1
Print Media: Banners, Information Board	Yes: 344	34.4
Health Brochures: Clinics or Hospitals	Yes: 408	40.8
Digital Media: Internet, Mobile phone	Yes: 516	51.6
Doctor / Nurse	Yes: 738	73.8
**Indicators**		
Obesity	Yes: 56	5.6
Diabetes	Yes: 383	38.3
Hypertension	Yes: 70	7.0
Kidney Stone	Yes: 29	29.0
**Laboratory Identification**		
Blood eGFR	Yes: 309	30.9
Urine albumin	Yes: 176	17.6
Scan ultrasound MRI/CT	Yes: 21	21.0
**Sign and Symptoms**		
Sleep Problem	Yes: 16	1.6
Swelling on lower limbs	Yes: 168	16.8
Dry and itchy skin	Yes: 17	1.7
Frequent urination	Yes: 51	5.1
Bloody urine	Yes: 37	3.7
Foaming urine	Yes: 60	6.0
Swelling around the eyes	Yes: 26	2.6
Lack of appetite	Yes: 16	1.6
Muscle cramps	Yes: 13	1.3
**CKD Practice Prevention**		
Self-health monitoring	Yes: 120	12.0
Balanced nutrition	Yes: 204	20.4
Health lifestyle	Yes: 245	24.5
Health providers	Yes: 93	9.3
Traditional Medicines	Yes: 9	0.9

However, 88.6% of respondents were also suffering from other illnesses aside from diabetics, with 64.1% monitoring their levels of glucose daily to ensure that their health remained good. Table 2 displays the overall understanding of diabetic characteristics, with more than 89% seemingly uncertain of the significance of clear glycosuria, ketonuria, and kidney stones. Summarizing respondent CKD-related resources of information, 45.2% gained CKD knowledge from mass media and 65.6% thought print media was not a positive learning tool. Furthermore, health brochures in clinics or hospitals played a role in distributing CKD knowledge to 40.8%, although approximately 48.4% disagreed with digital media, such as the internet and mobile phones. However, most agreed that physicians and healthcare workers, such as doctors and nurses, playing a big role in spreading CKD information.

### Univariable analysis on predictors variable toward CKD perception

The majority of respondents (74%) had a negative perception of CKD prevention. On bivariate analysis, factors correlating with poor perception toward CKD prevention included: sex (*p* < 0.001), occupation sector (*p* < 0.001), education level (*p* < 0.001), diseases other than diabetes (*p* < 0.001), high blood glucose level (*p* < 0.001), clear glycosuria (*p* < 0.001), ketonuria (*p* < 0.001), and renal stone (*p* < 0.001). Details can be seen in Tables 3 and 4. Most of the demographic characteristics were not significantly associated with poor CKD prevention.

**Table 3:** Bivariate analysis of socio-demographic and socio-economic factors associated with the perception of CKD prevention.

	Variable	Perception		χ^2^ value	*P*-value
		Poor	Good		
Age	> 60 years < 60 years	454 (74.1%) 285 (73.5%)	158 (25.9%) 103 (26.5%)	0.444	0.833
Gender	Male Female	549 (79.9%) 189 (60.4%)	138 (20.1%) 124 (39.6%)	42.145	< 0.001
Marital status	Married Others	734 (74.3%) 4 (0.0%)	254 (25.7%) 8 (0.0%)	8.937	0.030 0.001
Religion	Islam Buddha Hindu Christian	423 (74.6%) 283 (74.9%) 28 (60.9%) 4 (44.5%)	144 (25.4%) 95 (25.1%) 18 (39.1%) 5 (55.5%)	7.561	0.109
Race	Malay Chinese Indian	423 (74.7%) 283 (74.7%) 32 (58.9%)	143 (25.3%) 96(25.3%) 23 (41.1%)	6.881	0.076
Occupation sector	Government Private Business Self-employed Housewife/husband	15 (45.5%) 43 (67.2%) 77 (86.5%) 102 (94.4%) 501 (71.0%)	18 (54.5%) 21 (32.8%) 12 (13.5%) 6 (5.6%) 205 (29.0%)	56.023	< 0.001
Education level	Degree/Master/PhD Diploma/Certificate Secondary school Never attend school	26 (55.3%) 44 (62.9%) 610 (75.9%) 58 (73.4%)	21 (44.7%) 26 (37.1%) 194 (24.1%) 21 (26.6%)	14.426	0.002
Health Insurance	Yes No	140 (78.2%) 598 (72.8%)	39 (21.8%) 223 (27.2%)	2.195	0.138
Life Insurance	Yes No	140 (78.2%) 598 (72.8%)	39 (21.8%) 223 (27.2%)	2.195	0.138
Accident Insurance	Yes No	83 (82.2%) 655 (72.9%)	18 (17.8%) 244 (27.1%)	4.079	0.043

**Table 4:** Bivariate analysis of the predictors on general knowledge associated with the perception of CKD prevention.

	Variable	Perception		χ^2^ value	*P* value
		Poor	Good		
Do you have other diseases besides diabetes?	Yes No	677 (76.4%) 61 (53.5%)	209 (23.6%) 53 (46.5%)	27.399	< 0.001
Have you ever been told by a physician that you have diabetes?	Yes No	710 (73.6%) 28 (82.4%)	256 (26.4%) 6 (17.6%)	4.128	0.127
Duration of Diabetes	12.85 (7.2)	11.70 (7.9)	2.038*	0.042	
Are you trying to get health information?	Yes No	504 (76.0%) 234 (69.4%)	159 (24.0%) 103 (30.6%)	5.006	0.025**
Would you like to participate in a health program organized by the hospital?	Yes No	446 (77.0%) 292 (69.4%)	133 (23.0%) 129 (30.6%)	7.418	0.006***
Do you monitor glucose levels? Biochemistry Profile	Yes No	461 (71.9%) 277 (77.2%)	180 (28.1%) 82 (22.8%)	3.268	0.071
Glucose level	7.98 (2.71)	7.77 (2.78)	1.041*	0.298	
HbA1C level	7.7 (1.7)	7.8 (1.8)	0.884*	0.377	
Post prandial 2 h level	7.7 (2.1)	7.5 (2.1)	0.818*	0.414	

*Mean (Sd).

** < 0.05.

*** < 0.005.

Multiple logistic regression tests were conducted to evaluate predictor variables of health determinants that may influence the perceptions of CKD and its prevention. Table 5 shows the results based on multiple logistic regression analysis to test the model set by the determinant factors chosen. Three models were generated. Model 1 indicates the influence of sociodemographic factors on the perceptions of CKD and its prevention among respondents. This model classified 76.5% of the data with χ^2^ = 110.556. Model 2 illustrates the influence of knowledge on self-monitoring advocacy (clear glycosuria and sleep problems), knowledge of preventive behavior (types of mass media, print media, and digital media), and knowledge on treatment compliance (laboratory test identification; urine albumin), which is associated with poor CKD perception; the prevention model classified 76.7% of the data with χ^2^ = 107.0109, *p* < 0.001. Model 3 examined whether the combination with the previous factors and by adding the socio-psychological factors had any relationship with poor perception among respondents, with 81.3% of the data classified with χ^2^ = 65.902, *p* < 0.001.

**Table 5:** Multiple logistic regression for variables predicting poor perceptions of CKD prevention.

Variables	Model 1				Model 2				Model 3			
	B	S.E	AOR	95%CI	B	S.E	AOR	95% CI	B	S.E	AOR	95%CI
**Constant**	1.169	0.430	3.220		3.154	0.670	17.228		6.094	1.178	443.015	
**Socio-Demographic** **Occupation**												
Self-Employed	2.341	0.418	10.390	4.58–23.56	1.824	0.452	6.196	2.55–15.03	1.336	0.475	3.803	1.49–9.65
Housewife	0.990	0.343	2.692	1.37–5.26	0.856	0.380	2.353	1.11–4.95	0.609	0.402	1.838	0.83–4.03
Private	0.890	0.435	2.434	1.03–5.70	0.622	0.472	1.862	0.73–4.70	0.311	0.499	1.365	0.51–3.62
Government			1				1				1	
**Knowledge on:** **Self-Monitoring Advocacy** **Clear glycosuria**												
Weak					1.700	0.539	5.472	1.90–15.74	1.353	0.565	3.870	1.27–11.72
Adequate							1				1	
**Sleep Problem**												
Weak					1.766	0.789	5.847	1.24–27.46	1.790	0.817	5.991	1.20–29.68
Adequate							1				1	
**Preventive Behavior**												
**Print Media**												
Weak							1				1	
Adequate					1.199	0.220	3.317	2.15–5.10	1.032	0.228	2.807	1.79–4.39
**Digital Media**					0.833	0.175	2.300	1.63–.3.24	0.585	0.188	1.795	1.24–2.59
Weak							1				1	
Adequate												
**Treatment Compliance**												
Urine Albumin												
No					0.963	0.210	2.620	1.73–395	0.670	0.224	1.954	1.26–3.03
Yes							1				1	
**Perception**												
**Socio-Psychology** **Illness identity fear**												
Weak									0.804	0.183	2.235	1.56–3.19
Adequate											1	
**Medical Practice**									0.847	0.189	2.332	1.60–2.38
Weak											1	
Adequate												
**Co-operation**												
Weak									0.447	0.180	1.563	1.09–2.22
Adequate										1		
Coefficient Model -2 Log Likelihood Nagelkerk R Square Hosmer Lemeshow Test Classification overall percentage	χ^2^ = 110.556(3)*** 1039.721 0.153 *P* = 0.843 76.5				χ^2^ = 107.109(8)*** 932.613 0.286 *P* = 0.006 76.7				χ^2^ = 65.902(11)*** 866.711 0.361 *P* = 0.1 81.3			

*AOR*  Adjusted Odds Ratio, *CI*  Confidence Interval.

χ^2^ ( ) = Chi Square (df).

***p < 0.001 **p < 0.01 *p < 0.05.

Multiple logistic regression with backward logistics regression was applied.

Multicollinearity was checked and none found. No significant interaction variables were found.

Model 1 indicates the influence of sociodemographic factors on the perception of CKD and its prevention among respondents.

Model 2 illustrates the influence of self-monitoring advocacy, preventive behavior, and treatment compliance on the poor perception of CKD prevention.

Model 3 examined whether socio-psychological factors had a relationship with poor perception among respondents.

Model 1 indicates the influence of sociodemographic factors on the perception of CKD and its prevention among respondents. Only occupation showed a significant relationship with the regression model. Self-employment was 10 times more likely to relate to poor perception than government staff (95% CI 4.582–23.564, *p* < 0.001). Housewives were two times more likely to have a poor perception of CKD (95% CI 1.375–5.269, *p* < 0.001). Respondents who worked in a private agency were also two times (95% CI 1.038–5.707, *p* < 0.001) more likely to have a poor perception of CKD as opposed to government staff. This model provided good and acceptable outcomes (χ^2^(3) = 110.556, *p* < 0.001).

Model 2 illustrates the influence of self-monitoring advocacy, preventive behavior, and treatment compliance on the poor perception of CKD prevention. Respondents with partial awareness on self-monitoring advocacy on clear glycosuria were five times more likely to have poor perception than the other group (95% CI 1.901–15.749, *p* < 0.001). In contrast, those with knowledge on sleep-related problems was six times more likely to have a poor perception of CKD (95% CI 1.245–27.466, *p* < 0.001). Those who have received CKD information through preventive behavior such as print media were three times more likely to have a poor perception of CKD. In addition, the resources received were very less via digital media (AOR = 2.30; 95% CI 1.631–3.242, *p* < 0.001) and for treatment compliance, CKD laboratory test identification such as urine albumin (AOR = 2.62; 95% CI 1.737–3.2953, *p* < 0.001) were more likely to have a poor perception of CKD. Adding these five predictor variables significantly increased the fit of model 2 (χ^2^(8)= 107.109, *p* < 0.001) increased the predictive ability of CKD prevention compared to Model 1.

Model 3 shows several factors that have a significant influence on poor CKD perception. The model examined whether socio-psychological factors had a relationship with poor perception among respondents. Those who had weak illness identity fear were two times more likely to have poor perceptions (95% CI 1.563–3.196, *p* < 0.001). Respondents with weak medical practice (AOR = 2.33, 95% CI 1.609–2.381, *p* < 0.001) and weak cooperation (AOR = 1.563; 95% CI 1.099–2.224, *p* < 0.001) were more likely to have poor perceptions of CKD prevention. The additional predictor variables related to perception significantly improved the accuracy of the predictions compared to Model 2 (χ^2^(11) = 65.902, *p* < 0.001). Overall, multiple relationships were found in predicting the perception of CKD prevention, with 81% compared to the other models. Concerning the predictors of respondents’ poor perception, significant predictors were found to be associated with self-employed, housewives and househusbands, private, weak knowledge of clear glycosuria, sleep problems, print media, digital media, illness identity fear, weak medical practice, and weak co-operation factors. Details are listed in Table 5.

## Discussion

Improved perceptions of CKD prevention among the high-risk population necessitate a multifaceted approach that considers various aspects of human belief and behavior that cannot be summarized in a model based on measurable demographic, psychological, or social variables, such as age and gender. Despite the wide range of demographic characteristics considered in this study, the multilevel logistic regression model was unable to predict more than 40% of the outcomes regarding CKD preventive perceptions in the participants. A variety of subjective elements, including coping abilities, social expectations, personal beliefs, and views, will always affect CKD preventive behavior. The current study identification of determinants of CKD preventive views, on the other hand, may be valuable for the development of a model that may more precisely predict cancer screening uptake and, as a result, stimulate increased uptake.

In general, perceptions regarding CKD prevention and acceptance of its importance in terms of early identification continue to be inadequate. The CKDPS was evaluated in diabetic patients, and experts assessed the reliability and validity of the content to examine the scale together with developed guidance from collaboration with health care professionals. The items were tested for the final items in the pool on the clarity and relevance domains^10^. The CKDPS can be used to assess other high-risk groups to develop CKDs, such as obesity, hypertension, and cardiovascular disease, to assess their level of perception, and to take preventive measures. This study reported that the poor perception of the population at risk of developing CKD on how to prevent it revolved around socio-demographic, socio-economic, and general knowledge of self-monitoring advocacy, preventive behavior, and social-psychology issues.

This indicates that preventive behavior and treatment compliance factors were predictor variables that may influence poor perceptions of CKD prevention other than socio-demographic factors. Regarding socio-demographic aspects, it was found that occupation was one of the significant predictors. This study showed that occupation is closely related to poor understanding of CKD by diabetic patients because majority were housewives and pensioners that agreed to participate in this research. This study agrees with Boulware et al., who showed that occupation was significantly related to poor perception among patients with diabetes, who were associated with low perceived susceptibility to CKD^11^.

Those with higher education levels were more likely to have a good understanding of how to avoid CKD. Professionals/executives, younger individuals, and those with higher education levels and monthly income all had associated good knowledge scores concerning CKD. These findings are consistent with a study conducted among primary care patients in Singapore which revealed that professionals, those with younger ages, those having an above primary education level, and those with higher monthly household incomes were more likely to have better knowledge concerning CKD^12^.

There were two significant variables found in self-monitoring advocacy: clear glycosuria and sleep problems. Most patients did not know the meaning of the term’s clear glycosuria, ketonuria, or kidney stones. This was due to the term being commonly used by physicians or health care workers instead of diabetic patients. These are physical indicators that have been used to detect CKD among high-risk groups. Diabetic patients only regard their health status in terms of severity rather than knowing in-depth, their diseases based on medical reports. This is consistent with the findings of Yvette Brazier, stating since the symptoms of CKD can only be seen in the later stages, screening is recommended for high-risk groups. It is important for those at risk for developing kidney disease to regularly check their kidney function. Early detection can significantly help prevent serious kidney damage. The most common signs and symptoms of CKD, as discussed, are anemia, blood in the urine, high levels of glucose, ketonuria, kidney stones, and proteinuria^13^.

The majority of the respondents did not realize that sleep problems could be signs and symptoms of CKD. Sleep problems are one of the signs normally experienced by patients with diabetes. However, swelling around the eyes, lack of appetite, and muscle cramps were what most of the respondents had experienced. Health care providers tell the high-risk groups the signs and symptoms that they should look out for in order to diagnose problems and take early action to prevent CKD. The patient then needs to monitor for signs and report these during monthly check-ups. However, a study reported by The American Kidney Fund reported that swelling involving the lower limbs, frequent urination, and foaming urine are the most common signs indicating that a patient’s kidneys have started to fail^14^.

These findings are consistent with a study conducted by Platinga et al. 2012, showing that sleep problems were widespread more than 90% for any problem; averaging 10–40% for individual problems with diabetes, who were more likely to report multiple problems than those without diabetes^15^.

Almost half of the diabetic patients found out about CKD via print media. However, print media was not a useful resource among the respondents, while health brochures in clinics or hospitals did play a role in spreading CKD information, and some said digital media such as the Internet and mobile phones were not preferred resources respondents used to get information about CKD. This might be largely due to the elderly group’s lack of knowledge on the use of mobile phones. The respondents agreed that physicians, doctors, and nurses played a significant role in providing CKD information. According to Ng et al. (2017), respondents who had heard of CKD were more likely to have a better understanding of whether the information from newspapers, the Internet, medical personnel, or health campaigns were all associated with better knowledge about CKD^16^.

This is consistent with a study in a Canadian province that showed that awareness of kidney health was successfully increased after a public health campaign, especially among those with lower than high school education and low yearly income^17^. However, information from family or friends was not related to good knowledge via CKD scores. This suggests that existing health campaigns and the propagation of information about CKD through the mass media and official health authorities can be effective and successful in increasing knowledge about CKD among at-risk populations.

This study showed that 80% of the respondents were not familiar with the urine albumin test methods for CKD identification. Laboratory method diagnosis using a blood test on eGFR is one of the most reliable and familiar methods of CKD detection. The term in laboratory investigations such as urine albumin, eGFR, and MRI/CT scan was not familiar among diabetic patients and their knowledge is very limited. Meanwhile, diabetic patients only concern about their health status conditions rather than in terms of the severity rather than knowing in-depth regarding their diseases based on medical reports. These findings are consistent with a study conducted by NKF (2019), who reported that urine tests only showed comparable amounts of albumin, creatinine, and ACR. Furthermore, few researchers are aware that ultrasound scans can be used for CKD detection. The National Kidney Foundation (2019) stated that two tests, the albumin to creatinine ratio (ACR) and glomerular filtration rate (GFR), can measure kidney function and are performed through a blood test. The ACR test detects abnormalities through albumin reading in an early sign of kidney damage, and the GFR determines the stages of kidney disease. Furthermore, imaging tests, such as ultrasound and CT scans, can also be used to detect abnormalities in the size and position of the kidney^18^.

The last domain that contributed to this study was psychology. Three were three predictors found: illness identity fear, medical practice, and cooperation. In addition, diabetic respondents' understanding of CKD prevention practices in social psychology was higher than in other at-risk groups. Socio-psychological influences consist of concepts of illness and identity, coupled with low motivation, lack of awareness and accountability, fear of CKD, and poor medical practice. Those with weak illness identity fear were twice as likely to have poor perceptions.

These findings are consistent with the study conducted by Clarke et al., who also reported that most studies on CKD patients found that they were uncomfortable discussing their illness. Those who perceived that they had greater personal control were male, especially at the earlier stages of the disease. Confidence in their treatment positively influences patients' CKD perceptions regarding their condition, and the belief that continuing to follow medical advice is the best way to control their disease. In addition, moderately negative responses may be due to patient condition, since most CKD patients experience emotional distress even in the early stages of the disease^19^.

Respondents who had weak medical practice and weak cooperation were more likely to have a poor perception of CKD prevention in this study. Patients with CKD reported that they worried about their health. However, the levels of concern stated were comparable with a previous study in patients with ESRD requiring dialysis treatment, which also indicated that patients have some concerns regarding the negative effects of treatment that included concerns related to their potential future need for dialysis treatment. This is consistent with a previous study that identified additional patient concerns about medication side effects, including the belief that taking medication could affect their behavior^19^.

Meanwhile, the causal belief was not found in all the studies reviewed. The causes identified, such as aging, diet, genetics, stress, long-term use of medication, karma, and other chronic illnesses may be the result of cultural influences, gender, health education level, or stage of CKD progression. Causal beliefs have been shown to impact self-management behavior in long-term conditions^19^. Ryz et al. found that overall patient understanding of their illness increased from 7% pre-campaign messaging to 25% post-campaign messaging. Approximately two-thirds of their respondents correctly identified a big campaign message^17^.

This is consistent with a study conducted by Mahmoud Loghman (2003), who found many factors associated with poor compliance that have also been identified in several studies, such as frequent dosing, patient perception of treatment benefits, poor patient-physician communication, lack of motivation, poor socioeconomic background, lack of family and social support, and comparative youth^20^. Few strategies have been suggested to improve compliance with medication, and most are without scientific validation. Strategies to improve compliance in dialysis and transplant patients are similar to those prescribed for other chronic conditions, including simplifying the treatment regime, establishing a partnership with the patient, and increasing awareness through education and feedback^21^.

This study reported that cooperation was significantly different among diabetic patients, as assessed by many factors such as care, lifestyle, self-management, and government support to prevent CKD. The combination of predictors was accurate and beneficial compared with covering only one component.

## Strength and weakness of the study

This study had several strengths. First, it involved many respondents with a high level of feedback response. The large sample size provided a better picture of the overall population, reduced the effects of extreme or isolated points, and reduced the risk of bias. This study can help researchers make more meaningful assessments. The high response rate minimized the risk of biased responses and increased the likelihood of more accurate results.

In addition, this study used the strong and comprehensive CKDPS to measure CKD prevention in diabetic patients, producing good match accuracy in the validation process and high reliability (Anuar et al. 2020). The scale used was comprehensive in terms of perception measurement because it was built on a health belief model. It is also a new instrument specifically targeted at groups at high risk of developing CKD in the future. Therefore, it helped fill the knowledge gap in the study regarding how CKD prevention is perceived and understood. In addition, its high-reliability rating also indicates that the scale was well developed, as it successfully matched the study population. The measured perception values recorded were also more generally applicable and authentic.

The use of 10-point interval scales in the questionnaire was to measure perceptions; it allowed respondents to state their level of agreement with each question. The selection of sufficient interval scales enabled respondents to express their perceptions more openly and without limitation. In addition, this study was a quantitative study involving a large sample size to determine the responses to an issue. The findings were also suitable for extrapolation to a larger population and could also help to correct and support the smaller amount of information and data that could be obtained from a single focus group interview or discussion.

Apart from the strengths of this study, it also had some limitations. Although there were some limitations to this study, the objectives of this research were achieved. The limitations identified could serve as the basis for improvements in future studies. Because the study design was cross-sectional, the precise causes of the factors influencing the responses could not be determined. There were differences in the demographics of the respondents in this study, but these were not enough to represent the perceptions of all diabetics. In addition, the results of this study could underpin research with a larger sample size and a wider variety of frequencies to match demographic characteristics. Ethnicity and individual religious beliefs could also play a role in influencing an individual’s perceptions. The second limitation was related to the data. Perception measurement data were obtained from respondents only at one point in time. General knowledge could change perceptions, which could follow current trends. Changes in perception may influence the attitude toward chronic kidney disease in diabetic patients.

Other concerns that need to be considered as a broad range of tendencies for participants to respond inaccurately or falsely to questions are known by respondent bias. These biases are common in self-report participant studies, such as standardized interviews or surveys. Answer biases may affect the validity of questionnaires or surveys. Selection bias can occur when selecting participants in a study or their probability of being retained in the study contributes to a result that is different from researchers who had enrolled the entire target population.

Finally, the perceptions of researchers and patients may have been contradictory during the interview session. This is due to multiple factors, including patient education and awareness. Other factors, including age, contributed to these issues.

## Conclusion

Having weak physical knowledge of clear glycosuria, weak sleep issue knowledge, weak knowledge of useful print media services, digital media as preventive actions, no knowledge of urine albumin testing, weak perception of disease identity, medical practice and cooperation, and even occupation were significant predictors of poor perception expectations. CKD patient education programs must include specific target groups. The study instrument could be used to measure high-risk groups of patients, such as those with obesity, hypertension, and cardiovascular diseases, influencing their perception of CKD prevention. This study will lead to the development of CKD policy makers in Malaysia. As per future research guidelines, a cohort study, instead of a cross-sectional study design, was recommended. This approach applies to other organizations, such as routine new recruitment health checks and new student registrations. Media support is crucial for supporting and improving the positive view of CKD. This will improve individual interaction with different obstacles and disclosure, improvising this study to enhance individual behavioral responses to CKD prevention, indeed, indirectly.

## Data Availability

The data sets generated and analysed during the current study are available from the corresponding author on reasonable request.
